# Patient experiences receiving rehabilitation care *via* telehealth: Identifying opportunities for remote care

**DOI:** 10.3389/fresc.2023.1049554

**Published:** 2023-02-02

**Authors:** Jennifer G. Goldman, Douglas Merkitch, David Brewington, Hannah Peirce, Monica Rho, Prakash Jayabalan, Jessica Curran, Kimberly Brennan

**Affiliations:** ^1^ Parkinson's Disease and Movement Disorders, Shirley Ryan AbilityLab, Chicago, IL, United States; ^2^ Department of Physical Medicine and Rehabilitation, Northwestern University Feinberg School of Medicine, Chicago, IL, United States; ^3^ Department of Neurology, Northwestern University Feinberg School of Medicine, Chicago, IL, United States; ^4^ Information Systems, Enterprise Data Warehouse, Shirley Ryan AbilityLab, Chicago, IL, United States; ^5^ Outpatient Physical Therapy, Shirley Ryan AbilityLab, Chicago, IL, United States; ^6^ DayRehab®, Shirley Ryan AbilityLab, Chicago, IL, United States

**Keywords:** care coordination, COVID-19, interdisciplinary care, multidisciplinary care, neurorehabilitation, patient satisfaction, physiatry, telemedicine

## Abstract

Use of telehealth has grown substantially in recent times due to the COVID-19 pandemic. Remote care services may greatly benefit patients with disabilities; chronic conditions; and neurological, musculoskeletal, and pain disorders, thereby allowing continuity of rehabilitation care, reducing barriers such as transportation, and minimizing COVID-19 exposure. In March 2020, our rehabilitation hospital, Shirley Ryan AbilityLab, launched a HIPAA-compliant telemedicine program for outpatient and day rehabilitation clinics and telerehabilitation therapy programs. The objective of this study was to examine patients' experiences and satisfaction with telemedicine in the rehabilitation physician practice, including novel virtual multidisciplinary evaluations. The present study examines survey data collected from 157 patients receiving telemedicine services at Shirley Ryan AbilityLab from December 2020–August 2021. Respondents were 61.8% female, predominantly White (82.2%) with ages ranging across the lifespan (69.4% over age 50 years). Diagnostic categories of the respondents included: musculoskeletal conditions 28%, chronic pain 22.3%, localized pain 10.2%, neurological conditions 26.8%, and Parkinson's and movement disorders 12.7%. Survey responses indicate that the telemedicine experiences were positive and well received. The majority of participants found these services easy to use, effective, and safe, and were overall satisfied with the attention and care they received from the providers—even for those who had not previously used telehealth. Respondents identified a variety of benefits, including alleviating financial and travel-related burdens. There were no significant differences in telehealth experiences or satisfaction across the different clinical diagnostic groups. Respondents viewed the integrated physician and rehabilitation therapist telehealth multidisciplinary model favorably, citing positive feedback regarding receiving multiple perspectives and recommendations, feeling like an integrated member of their healthcare team, and having a comprehensive, holistic team approach along with effective communication. These findings support that telemedicine can provide an effective care model in physiatry (physical medicine and rehabilitation) clinics, across different neurological, musculoskeletal, and pain conditions and in multidisciplinary team care settings. The insights provided by the present study expand our understanding of patient experiences with remote care frameworks for rehabilitation care, while controlling for institutional variation, and ultimately will help provide guidance regarding longer term integration of telemedicine in physiatry and multidisciplinary care models.

## Introduction

The use of telehealth has gained increasing interest in clinical care and research due to evolving patient needs for telemedicine and telerehabilitation as a result of the COVID-19 pandemic and the growing integration of technology into care models (1–4). While telehealth technology has been utilized over the years in different disciplines (e.g., medicine, neurology, radiology) to bring care to populations and underserved areas beyond traditional medical centers, the use of telehealth in physiatry clinical practice and for delivering rehabilitation services has been less frequently used or studied, especially prior to the COVID-19 pandemic (2, 3, 5). In a report by the Agency for Healthcare Research and Quality report in June 2016, systematic reviews provided moderate evidence and potential benefit of delivering telerehabilitation care for cardiovascular disorders and other conditions treated in the field Physical Medicine and Rehabilitation (PM&R) (6). However, the pandemic changed the landscape of virtual care with markedly increased implementation of telemedicine in rehabilitation (physiatry, PM&R) practices and telerehabilitation delivering physical, occupational, and speech therapy services. The use of telemedicine for physiatry may be particularly helpful to improve access to care for patients with disabilities, neurological disorders, mobility or movement-related issues, and chronic conditions that would traditionally require physical travel to appointments, frequent utilization of healthcare services, increased need for subspecialty medical care, and coordination of care across different care disciplines. Furthermore, telemedicine for physiatry would be beneficial to minimize COVID-19 exposure risk in often medically vulnerable populations. Since the onset of COVID-19 in 2020, several studies describe the benefits of telemedicine in rehabilitation populations and patient and/or provider satisfaction, primarily for outpatient sports and musculoskeletal issues in adults and children (7–14), spinal cord injury (15–17), cancer rehabilitation (18), and chronic pain (19–21). With the COVID-19 pandemic, there was a growing need for medical centers to convert traditional, in-person patient encounters to telehealth formats. Published reports document this change in outpatient and inpatient rehabilitation settings and various locations, e.g., United States, Europe, and Asia (7–22). To date, less is known about the benefits or experiences in physiatry practices for other neurological conditions, in coordinated multidisciplinary team settings, or in comparisons across different clinical rehabilitation populations.

On March 30, 2020, in response to the COVID-19 public health emergency, our rehabilitation hospital, Shirley Ryan AbilityLab, launched a HIPAA-compliant telehealth program for outpatient and day rehabilitation program physician practices and rehabilitation therapies. Shirley Ryan AbilityLab is one of the leading rehabilitation institutions in the United States, providing care at the inpatient rehabilitation hospital facility in downtown Chicago, IL and across a broad network of outpatient and day rehabilitation (DayRehab®) locations throughout the Greater Chicagoland area. In addition to the inpatient facility, the organization provides care to adult neurological, adult orthopedic, and pediatric patients in outpatient clinics, a pain management center with multidisciplinary care, and at day rehabilitation (DayRehab®) programs that treat a variety of neurological conditions throughout the Chicago area. The feasibility and success of a rapid shift across outpatient and DayRehab® programs to a telerehabilitation model for physical, occupational, and speech therapy services using a secure, HIPAA-compliant, Cisco Webex platform have been demonstrated by Brennan et al. (23) Similarly, the Shirley Ryan AbilityLab physician clinics underwent a swift transition from entirely in-person clinic appointments to exclusively telemedicine in March 2020, with a hybrid model available for either in-person or telehealth starting in June 2020. These virtual telemedicine physician visits occurred for patients in the outpatient and DayRehab® settings, with a subset receiving virtual multi- or inter-disciplinary care (i.e., physician/advanced practice provider, nurse, physical therapy, occupational therapy, and speech language pathology delivering care together) in the Pain Management and Parkinson's disease and Movement Disorders programs. These remote care visits allowed for video and audio communication to deliver patient care and thereby maintain continuity of care and provide new evaluations during the pandemic. As many of our rehabilitation populations were also at high risk for COVID-19 and its effects due to the nature of their underlying condition, these services permitted access to care while maintaining social distance and reducing risk of infection exposure and spread. As such, the Shirley Ryan AbilityLab telehealth program provides an opportunity to examine telehealth delivery models across multiple service lines and disciplines, as well as across diverse patient populations and conditions, all integrated within the same organization.

While our knowledge and experience with telehealth in rehabilitation settings and populations has grown substantially since March 2020, there remain unanswered questions regarding the experience of the patient and healthcare provider with telehealth and the optimal delivery methods of telehealth for different rehabilitation patient populations and across the age spectrum. The overall goal of this study was to better understand patients' perceptions, attitudes, and experiences with telehealth services at Shirley Ryan AbilityLab, particularly since this was a new service that was not previously offered at the organization prior to the onset of the COVID-19 pandemic. We also sought to determine whether patients with different conditions (e.g., pain, musculoskeletal, neurological) who may have distinct rehabilitation needs varied in their experiences with telehealth services. Lastly, we examined the effectiveness and satisfaction of delivering multidisciplinary care (physician and rehabilitation therapists) *via* telehealth. This information will help to inform future telehealth use, continued quality improvement and development of best practices, and ultimately, the ability to examine effects on patient outcomes.

## Materials and methods

### Study design

***Survey development:*** Survey questions were developed by an interdisciplinary team of physicians, allied health therapists, administrators, researchers and data scientists (J.G.G., M.R., *P*.J., J.C., K.B., D.M., D.B.) at our rehabilitation hospital. The survey questions assessed the perceptions, attitudes, experiences, and satisfaction of those receiving virtual care across a variety of domains, including effectiveness of achieving objectives, comfort, attention and care quality from providers, safety, and privacy. Survey responses utilized Likert scales, multiple choice questions, and free text, where appropriate (Supplementary Material). The survey was reviewed and tested prior to distribution by the authors and colleagues (i.e., nurse practitioner, physician, and physical therapist) who were not involved in question development.

***Setting and participants:*** Surveys were distributed electronically between December 17, 2020 through August 12, 2021 to patients who received telemedicine services identified through the rehabilitation hospital's Enterprise Data Warehouse (EDW), beginning March 30, 2020 through July 31, 2021. These patients included those seen for telemedicine evaluation and management by adult and pediatric rehabilitation physicians or advanced practice providers in the physician practice's outpatient rehabilitation clinics and DayRehab® program at Shirley Ryan AbilityLab. A subset of the telemedicine encounters included multidisciplinary care visits in which evaluations included not only the physician, but also allied health therapists (occupational therapy, physical therapy, speech language pathology) in the same visit; this type of visit occurred specifically in new patient and follow up evaluations in the pain management center performed asynchronously (i.e., multidisciplinary: physician and therapists in separate virtual settings for the appointment) and for only new patient evaluations in the Parkinson's disease and movement disorders clinic performed synchronously (i.e., interdisciplinary: therapists and physician in the same virtual setting at the same time) (24, 25). The survey included an optional section to capture telehealth experiences of these multidisciplinary care clinics.

All patients who received telemedicine services in this timeframe received an email invitation for the survey and a link to the informed consent document. Participants were limited to those residing in the United States or United States territories and able to be consented in English. For participants who were minors, parents were included in the research, consenting process, and data collection. Recruitment methods included email invitations, posted flyers in the clinics and hospital, discussions with medical and rehabilitation staff, and potential participants also received automated reminders *via* email for the survey after the initial email invitation.

Survey data was collected *via* the secure web application, Research Electronic Data Capture (REDCap), which is a secure, web-based software platform designed to support data capture for research studies (26). The study was approved by the Northwestern University Institutional Regulatory Board.

### Statistical methods and data analysis

Data for participant diagnoses were based on ICD-10 codes entered by the provider completing the telemedicine visit. Primary diagnoses, and where noted, secondary and tertiary diagnoses were reviewed from the linked EDW data output and electronic health record for categorization. Diagnoses were grouped into the following clinical categories: 1) Musculoskeletal conditions (e.g., spondylotic disorders, tendonitis, scoliosis, rheumatological disorders, or neck or back pain that was not chronic), 2) Chronic pain (e.g., chronic face, neck, back or other body region pain specifically denoted as chronic), 3) Localized pain (e.g., non-chronic pain with specific extremity or joint region noted), 4) Neurological conditions (e.g., brain injury, spinal cord injury, peripheral nerve disorders, cerebral palsy, stroke, multiple sclerosis), and 5) Parkinson's and movement disorders (e.g., Parkinson's disease, Lewy body dementia, Huntington's disease).

Data for survey responses was analyzed using SPSS version 28 software. Data was analyzed with descriptive statistics and for the diagnostic group comparisons using non-parametric statistics with Chi-square and Kruskal-Wallis, with post-hoc comparisons where appropriate. Statistical significance was set at *p* < 0.01 for diagnostic group comparisons.

## Results

### Survey responses and characteristics of respondents

The survey was distributed to 2,514 individual patients representing 4,618 patient encounters. Data is presented for 157 unique participant survey responses from this distribution (6.3%), representing the initial telehealth encounter at either the outpatient or day rehabilitation physician practices. Survey respondents had telehealth visits that occurred from May 2020 through July 2021, with over 87% of the responses from the survey invitation launch occurring between November 2020 to July 2021 (5.1% of respondents had visits from May to October 2020). A total of 55 telehealth visits occurred in 2020 and 102 telehealth visits, in 2021.

Characteristics of the survey respondents are presented in Table 1. The survey respondents (*n* = 157) were 61.8% female, predominantly White (82.2%, with 7.6% Black or African American, 2.5% Asian); most respondents were not Hispanic or Latino (92.8%). Ages ranged from 3 to 7 years to 80–89 years old with the majority over the age of 50 years (69.4%) and only 2.5% under the age of 18 years. The majority of respondents had a college or graduate degree (74.6%: Bachelor's/college 33.8% and graduate degree 40.8%, respectively), with 22.3% having either high school degree, some college but no degree, or an Associate's degree. The distance from the respondents' home to their Shirley Ryan AbilityLab patient care location was less than 5 miles for 46.8%, between 11 and 20 miles for 19.9%, and greater than 20 miles for 33.3%. Insurance providers for the respondents included: commercial insurers (52.6%), Medicare (41%), Medicaid (4.5%), or other/preferred not to answer (1.9%). The provider visits represented primarily follow up visits (86.2%) with 13.8% as new patient evaluations; the telehealth encounters represented the outpatient physician clinics in 98.1% of responses.

**Table 1:** Sample characteristics by telehealth usage.

Characteristic	*n* = 157
**Age, *n* (%)**	
<18 years	4 (2.5)
18–49 years	44 (28)
50–69 years	71 (45.2)
70–89 years	38 (24.2)
**Female, *n* (%)**	97 (61.8)
**Race/Ethnicity, *n* (%)**	
White	129 (82.2)
Black/African American	12 (7.6)
Asian	4 (2.5)
Native American or American Indian	0
Native Hawaiian or Pacific Islander	0
Other	4 (2.5)
Prefer not to answer	7 (4.5)
**Education, *n* (%)**	
Less than high school	5 (3.2)
High school degree	7 (4.5)
Some college but no degree	20 (12.7)
Associate degree	8 (5.1)
Bachelor's (college) degree	53 (33.8)
Graduate	64 (40.8)
Distance, *n* (%)*	
0–5 miles	46 (29.5)
6–10 miles	27 (17.3)
11–15 miles	22 (14.1)
16–20 miles	9 (5.8)
Greater than 20 miles	52 (33.3)
Insurance provider, *n* (%)*	
Medicare	64 (41)
Medicaid	7 (4.5)
Commercial Insurer	82 (52.6)
Prefer not to answer	3 (1.9)
**Technology used for telehealth, *n* (%)**	
Computer	69 (43.9)
iPad/tablet	33 (21.0)
Smart phone	29 (18.5)
Prefer not to answer	26 (16.6)

Results reported for *n* = 157.

*Sample *n* = 156.

**Figure 1:**
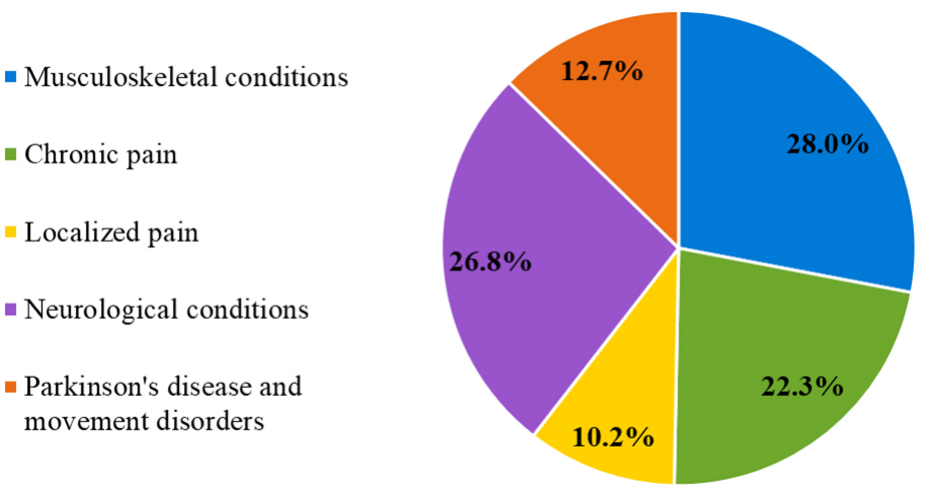
Survey respondents categorized by diagnostic group.

Regarding the diagnoses of respondents, diagnostic categories were as follows: musculoskeletal conditions 28%, chronic pain 22.3%, localized pain 10.2%, neurological conditions 26.8%, and Parkinson's disease and movement disorders 12.7% (Figure 1).

### Telehealth past and current experience

The majority of respondents had not previously utilized telehealth services for their care at Shirley Ryan AbilityLab (66.4%), but 61.6% of respondents had used telehealth services for care at other hospitals or medical centers and 84.9% used videoconferencing for other purposes such as work or with family and friends. Of those who previously used telehealth services at Shirley Ryan AbilityLab (*n* = 49), prior use was for other physician appointments (57.1%), telerehabilitation therapy appointments (14.3%) or both (20.5%). Regarding videoconferencing in general, respondents (*n* = 129) rated their comfort with use as “very comfortable” in 72.1%, followed by “somewhat comfortable” (15.9%), “neutral” (5.7%), or “somewhat uncomfortable” (1.3%).

Of the survey respondents, the majority completed the visit using videoconferencing (86.2%) with 13.8% utilizing phone only (no video). Of those using videoconferencing (*n* = 131), most used a computer system (52.7%), followed by an iPad or tablet (25.2%) or smartphone (22.1%).

**Figure 2:**
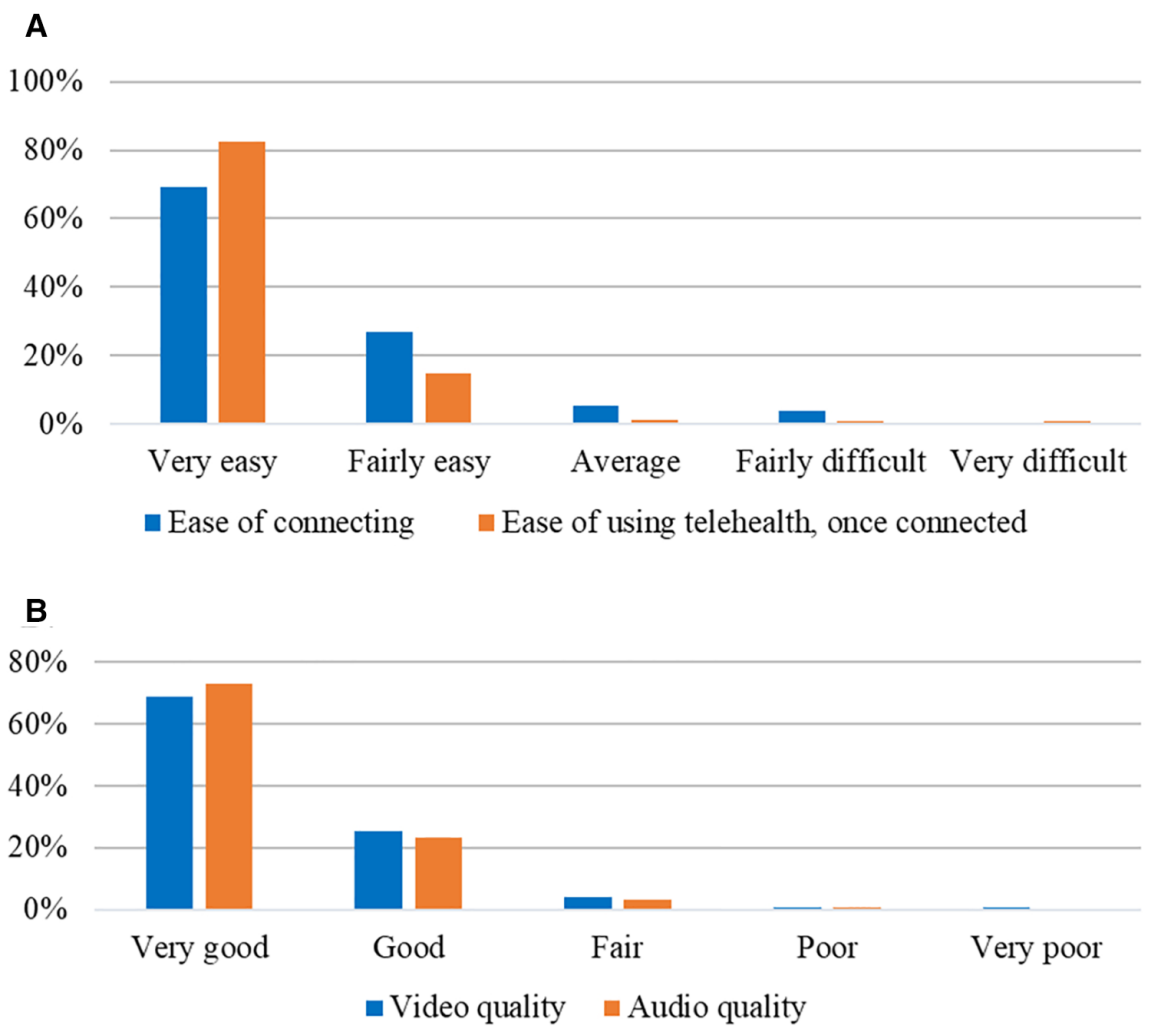
Ease and quality of telehealth experience.

Regarding the specific telehealth encounter and platform, respondents rated their ease of connecting to the visit as “very easy” in 69.1%, “fairly easy” (21.7%), “average” (5.3%), or “fairly difficult” (3.9%) with no one reporting connecting as “very difficult.” Once connected, the ease of using telehealth system was deemed “very easy” by 82.6% with only 2.7% reporting this as “average, fairly difficult or very difficult.” A small percentage (8.9%) had help from someone in their location during the telehealth visits, e.g., from a spouse/partner, parent, child or other family member or care giver. The majority of respondents rated the audio quality and video quality as “very good” in 72.8% and 69% and “good” in 23.2% and 25.4%, respectively (Figure 2).

### Effectiveness and satisfaction with telehealth visit

Ratings for the effectiveness and satisfaction of telehealth visits were overall positive and with high degrees of satisfaction for the full cohort (Table 2). The effectiveness of getting care *via* telehealth was rated as “very satisfied” in 75% of respondents, with less than 5% reporting being “somewhat dissatisfied or very dissatisfied.” The majority reported feeling comfortable in being evaluated and treated *via* telehealth as “very satisfied” in 68.7% and “somewhat satisfied” in 18%. Attitudes towards physical safety (e.g., moving, walking) during the telehealth visit and for privacy of the visit were highly regarded with ratings of “very satisfied” in 88.2% and 87.5%, respectively. Respondents also felt that they were able to explain and demonstrate their symptoms during telehealth (“very satisfied” in 71.7%) and to do so without the help of someone else (e.g., family member, caregiver) with “very satisfied” reported in 86.1%. Furthermore, the respondents felt that the provider was able to pay full attention to them, complete their assessments, and give necessary recommendations *via* telehealth (“very satisfied” in 86.1%, 70.9%, and 77.5%, respectively). Overall levels of satisfaction with the telehealth experience were high with 79.5% reporting “very satisfied” and only 3.3% being “somewhat dissatisfied or very dissatisfied.”

**Table 2:** Patient-reported experience, satisfaction, and effectiveness of telehealth.

	Very satisfied	Somewhat satisfied	Neutral	Somewhat dissatisfied	Very dissatisfied
Effectiveness of getting care through telehealth	75.0%	17.1%	3.9%	2.6%	1.3%
Comfort level in being evaluated and treated through telehealth*	68.7%	18.0%	9.3%	2.7%	1.3%
Feeling of physical safety during telehealth visit	88.2%	3.9%	5.3%	0.7%	2.0%
Feeling that your privacy was safe during the telehealth visit	87.5%	8.6%	2.6%	1.3%	0%
Overall satisfaction**	79.5%	15.2%	2.0%	1.3%	2.0%
Ability to explain and demonstrate symptoms	71.7%	21.1%	2.6%	2.6%	2.0%
Ability of provider to pay full attention to patient**	86.1%	11.9%	1.3%	0%	0.7%
Ability of provider to complete assessments**	70.9%	18.5%	5.3%	4.0%	1.3%
Ability of provider to give necessary recommendations**	77.5%	15.9%	3.3%	2.0%	1.3%
Ability to complete assessments without help^	86.1%	5.8%	4.4%	1.5%	2.2%

Results reported for *n* = 152.

*Sample n = 150.

**Sample *n* = 151.

^Sample *n* = 137.

The participants reported that their experience with telehealth was “better than expected” in 54.3% but “about the same as expected” in 45%; less than 1% (0.7%) reported that it was “worse than expected.” Regarding continuation of telehealth visits, if available as part of routine care, even after the COVID-19 pandemic, the majority stated that they would be “very likely” to continue this (58.6%) but 27.5% were “somewhat likely” and 13.8% were “neutral, somewhat unlikely or very unlikely” to do so. Recommendations for telehealth to other healthcare providers or friends or relatives were “very likely” in the majority (57.7% and 56.3%, respectively) but “somewhat likely” in 22.8% and 27.2%, “neutral” in 13.4% and 13.2%, and “somewhat unlikely or very unlikely” in 6.9% and 3.3%, respectively.

**Figure 3:**
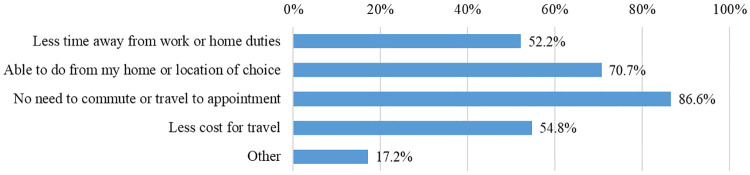
Perceived benefits of telehealth.

Of 151 respondents, 92.1% had received in-person clinical care for their condition in the past at Shirley Ryan AbilityLab. Compared to their in-person experiences, the survey respondents rated that the telehealth experience was “much more effective or somewhat more effective” in 40.3%, “equally effective” in 46%, and “somewhat less effective or much less effective” in 13.6%. A variety of benefits of telehealth were endorsed including reduced travel needs, burden (e.g., with pain, discomfort, mobility issues) and associated costs, less time spent away from work or home, ability to get earlier or sooner appointments with physician, and feeling safer due to COVID-19 concerns (Figure 3). The majority (71.3%) did not think that there were changes needed to the telehealth visit to make their experience better or more effective. However, a small percentage of respondents (1.9%–5.7%) cited that better audio or video connection, more space in their physical location at the time of visit, and assistance by family member or caregiver during the session would be helpful (Table 3).

**Table 3:** Patient-reported ways to make their telehealth visit better or more effective.

Item	*n* (%)
No changes needed - my telehealth visit went well	112 (71.3%)
Better audio connection	7 (4.5%)
Better video connection	9 (5.7%)
More space available in my location to demonstrate my issue to the provider (physician or therapist)	8 (5.1%)
Family member or caregiver to help me during the session (e.g., to position phone, computer camera)	3 (1.9%)
Other	18 (11.5%)

### Comparisons of telehealth experiences across diagnostic categories

Characteristics of the groups revealed that the majority of respondents were White and non-Hispanic or Latino (ranging from 74.3%–100% and 87.9%–93.8%, respectively). There was a female predominance across these groups (52.4%–75%) though this distribution was more evenly split for females/males in the Parkinson's and movement disorders (55%) and other neurological conditions (52.4%) diagnostic categories. There were no significant differences across the groups in race or ethnicity. There were significant diagnostic group differences with respect to age (H (4) = 18.26, *p* = .001) with post-hoc comparisons revealing the Parkinson's disease and movement disorders group being older than the other neurological conditions group (*p* = .005) and the localized pain group (*p* = .049), and the musculoskeletal group being older than the other neurological condition group (*p* = .021). As expected, due to disease characteristics of Parkinson's disease and Lewy body dementia, 95% of respondents in this diagnostic group were age 50 years or older. Although small numbers, only those with chronic pain or other neurological conditions included respondents under the age of 18 years (2.9% chronic pain; 7.2% neurological conditions which included some patients with cerebral palsy, spina bifida, and developmental disorders).

**Figure 4:**
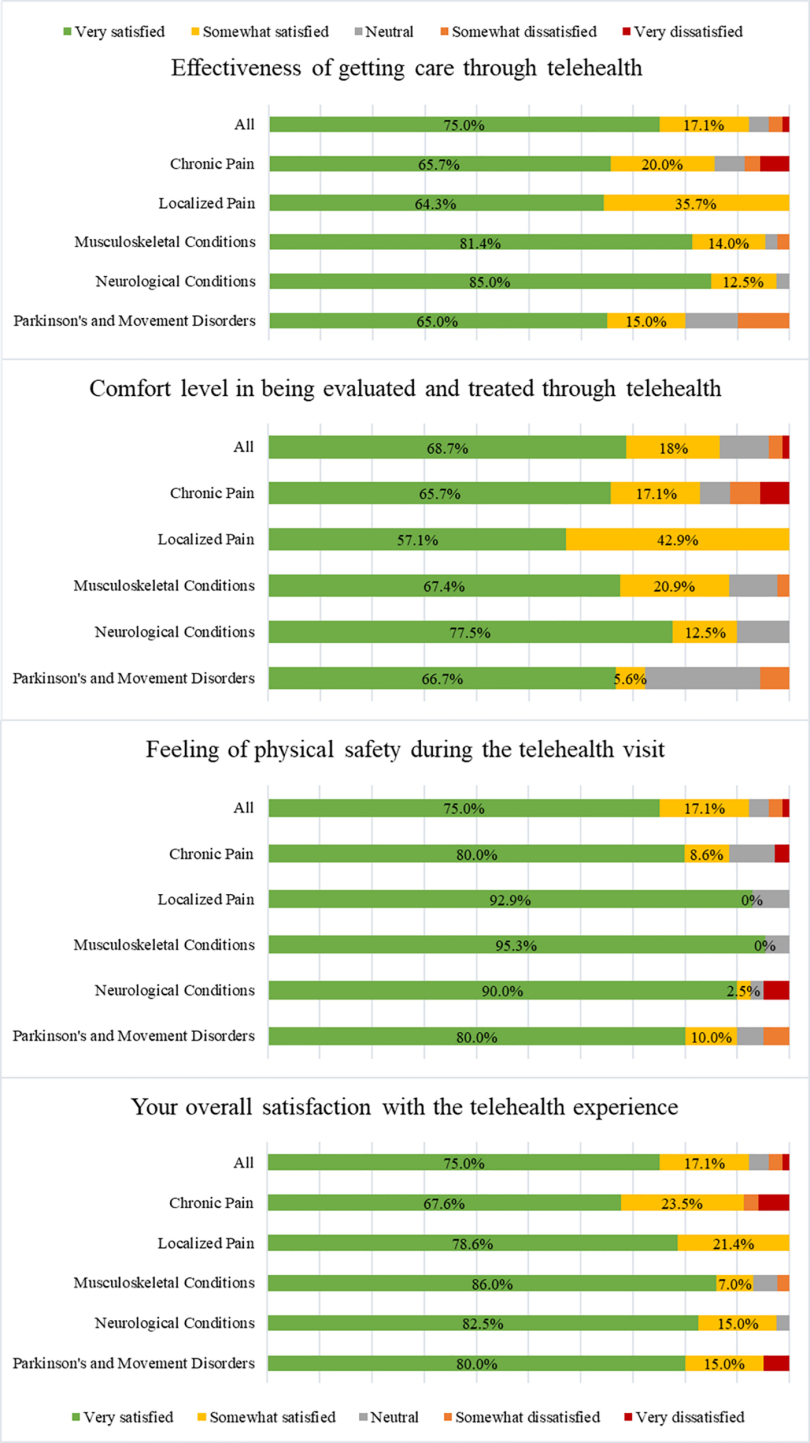
Experience, satisfaction, and effectiveness of telehealth by diagnostic category.

The majority of respondents in each diagnostic group rated their telehealth experiences as “very satisfied,” (Figure 4) though there was a broader distribution of responses across the Likert scales across these subgroups. Respondents across the diagnostic categories also did not differ significantly from each other regarding their experiences, attitudes, perceived benefits, or ratings (Table 4). There were no statistically significant differences across the clinical diagnostic categories regarding their comfort or ease of using telehealth services.

**Table 4:** Experiences reported across diagnostic groups during telehealth visits.

		Diagnostic groups					Statistical comparisons		
Survey item	Likert scale	Chronic pain	Localized pain	Musculo-skeletal conditions	Neurological conditions	Parkinson's disease and movement disorders	Kruskal-Wallis Test	df	*p* value
		*n* (%)	*n* (%)	*n* (%)	*n* (%)	*n* (%)			
Effectiveness of getting your care through telehealth	Very satisfied Somewhat satisfied Neutral Somewhat dissatisfied Very dissatisfied	23 (65.7) 7 (20.0) 2 (5.7) 1 (2.9) 2 (5.7)	9 (64.3) 5 (35.7) 0 0 0	35 (81.4) 6 (14.0) 1 (2.3) 1 (2.3) 0	34 (85.0) 5 (12.5) 1 (2.5) 0 0	13 (65.0) 3 (15.0) 2 (10.0) 2 (10.0) 0	7.314	4	.120
Comfort level in being evaluated and treated through telehealth*	Very satisfied Somewhat satisfied Neutral Somewhat dissatisfied Very dissatisfied	23 (65.7) 6 (17.1) 2 (5.7) 2 (5.7) 2 (5.7)	8 (57.1) 6 (42.9) 0 0 0	29 (67.4) 9 (20.9) 4 (9.3) 1 (2.3) 0	31 (77.5) 5 (12.5) 4 (10.0) 0 0	12 (66.7) 1 (5.6) 4 (22.2) 1 (5.6) 0	2.297	4	.681
Feeling of physical safety (e.g., moving, walking) during the telehealth visit	Very satisfied Somewhat satisfied Neutral Somewhat dissatisfied Very dissatisfied	28 (80.0) 3 (8.6) 3 (8.6) 0 1 (2.9)	13 (92.9) 0 1 (7.1) 0 0	41 (95.4) 0 2 (4.7) 0 0	36 (90.0) 1 (2.5) 1 (2.5) 0 2 (5.0)	16 (80.0) 2 (10.0) 1 (5.0) 1 (5.0) 0	5.646	4	.227
Feeling that your privacy was safe during the telehealth visit	Very satisfied Somewhat satisfied Neutral Somewhat dissatisfied Very dissatisfied	30 (85.7) 3 (8.6) 2 (5.7) 0 0	12 (85.7) 2 (14.3) 0 0 0	40 (93.0) 2 (4.7) 1 (2.3) 0 0	36 (90.0) 4 (10.0) 0 0 0	15 (75.0) 2 (10.0) 1 (5.0) 2 (10.0) 0	4.965	4	.291
Your ability to explain and demonstrate your symptoms adequately during telehealth	Very satisfied Somewhat satisfied Neutral Somewhat dissatisfied Very dissatisfied	24 (68.6) 6 (17.1) 3 (8.6) 0 2 (5.7)	9 (64.3) 3 (21.4) 0 2 (14.3) 0	32 (74.4) 9 (20.9) 1 (2.3) 1 (2.3) 0	33 (82.5) 7 (17.5) 0 0 0	11 (55.0) 7 (35.0) 0 1 (5.0) 1 (5.0)	6.579	4	.160
The ability of the provider to pay full attention to you during the telehealth visit**	Very satisfied Somewhat satisfied Neutral Somewhat dissatisfied Very dissatisfied	25 (71.4) 8 (22.9) 1 (2.9) 0 1 (2.9)	12 (85.7) 1 (7.1) 1 (7.1) 0 0	40 (93.0) 3 (7.0) 0 0 0	37 (92.5) 3 (7.5) 0 0 0	16 (84.2) 3 (15.8) 0 0 0	9.690	4	.046
The ability of the provider to complete his or her assessments for your condition with telehealth**	Very satisfied Somewhat satisfied Neutral Somewhat dissatisfied Very dissatisfied	21 (60.0) 9 (25.7) 1 (2.9) 2 (5.7) 2 (5.7)	10 (71.4) 3 (21.4) 0 1 (7.1) 0	33 (78.6) 5 (11.9) 2 (4.8) 2 (4.8) 0	31 (77.5) 5 (12.5) 4 (10.0) 0 0	12 (60.0) 6 (30.0) 1 (5.0) 1 (5.0) 0	4.832	4	.305
The ability of the provider to give you the necessary recommendations for my condition through telehealth**	Very satisfied Somewhat satisfied Neutral Somewhat dissatisfied Very dissatisfied	24 (68.6) 8 (22.9) 1 (2.9) 1 (2.9) 1 (2.9)	12 (85.7) 1 (7.1) 1 (7.1) 0 0	33 (78.6) 7 (16.7) 0 1 (2.4) 1 (2.4)	34 (85.0) 3 (7.5) 3 (7.5) 0 0	14 (70.0) 5 (25.0) 0 1 (5.0) 0	3.694	4	.449
Your ability to complete providers assessments without someone else (e.g., family member, caregiver) present to help you with the exam^	Very satisfied Somewhat satisfied Neutral Somewhat dissatisfied Very dissatisfied	23 (74.2) 6 (19.4) 2 (6.5) 0 0	12 (92.3) 0 1 (7.7) 0 0	37 (90.2) 0 2 (4.9) 0 2 (4.9)	32 (91.4) 1 (2.9) 1 (2.9) 0 1 (2.9)	14 (82.4) 1 (5.9) 0 2 (11.8) 0	4.636	4	.327
Your overall level of satisfaction with the telehealth experience**	Very satisfied Somewhat satisfied Neutral Somewhat dissatisfied Very dissatisfied	23 (67.7) 8 (23.5) 0 1 (2.9) 2 (5.9)	11 (78.6) 3 (21.4) 0 0 0	37 (86.1) 3 (7.0) 2 (4.7) 1 (2.3) 0	33 (82.5) 6 (15.0) 1 (2.5) 0 0	16 (80.0) 3 (15.0) 0 0 1 (5.0)	4.254	4	.373

Results reported for *n* = 152.

*Sample *n* = 150.

**Sample *n* = 151.

^Sample *n* = 137.

Statistical significance set at *p* < 0.01 for diagnostic group comparisons.

### Multidisciplinary care models *via* telehealth

Of all survey respondents, 25 participants identified that they received multidisciplinary care *via* telehealth and responded to the subset of questions regarding this care model. Similar to the demographics of the whole survey group, these respondents were primarily female (56%), over the age of 50 years (80%), White (80%), and not Hispanic or Latino (95.8%). Diagnostic categories for these visits included the following: Parkinson's and movement disorders (36%), neurological conditions (20%), musculoskeletal conditions (24%), chronic pain (16%), and localized pain (4%).

Respondents viewed the integrated physician/advanced practice provider and rehabilitation therapist telehealth team care model favorably (Table 5). These benefits were evident by the majority “strongly agreeing” that they received multiple perspectives and recommendations (68%), only needed to explain their concerns once (66.7%), felt like an integrated member of their healthcare team (80%), appreciated the comprehensive and holistic team approach (76%), and effectively addressed physical symptoms (62.5%) and mental health symptoms (56%). The respondents additionally “strongly agreed” that the team communication with each other was effective (76%) and also as communication directed with the patient (81.8%). This coordinated team care model allowed them to address topics that are not always covered in single provider visits (“strongly agreed” reported by 62.5%).

**Table 5:** Experiences with the multidisciplinary telehealth clinic model.

	Strongly agree	Somewhat agree	Neutral	Somewhat disagree	Strongly disagree
I received multiple perspectives and recommendations for my symptoms.	68.0%	20.0%	12.0%	0%	0%
I only needed to explain my concerns once.*	66.7%	29.2%	4.2%	0%	0%
I felt like an integrated member of my healthcare team.	80.0%	16.0%	0%	4.0%	0%
I appreciated the team's comprehensive and holistic approach to my condition.	76.0%	20.0%	4.0%	0%	0%
My concerns about physical symptoms were effectively addressed by the team.*	62.5%	25.0%	12.5%	0%	0%
My concerns about mental health symptoms were effectively addressed by the team.	56.0%	28.0%	12.0%	0%	4.0%
I felt like the team members effectively communicated with each other.	76.0%	8.0%	12.0%	0%	4.0%
I felt like the team members effectively communicated their findings and recommendations to me.**	81.8%	4.5%	9.1%	0%	4.5%
The time spent with each discipline was adequate.*	70.8%	16.7%	8.3%	0.0%	4.2%
The team approach allowed me to address topics that are not always covered in my visits with a single physician.	62.5%	20.8%	12.5%	0.0%	4.2%
This type of interdisciplinary team evaluation worked well in a telehealth format.	72.0%	12.0%	16.0%	0.0%	0.0%

Results reported for *n* = 25.

*Sample *n* = 24.

**Sample *n* = 22.

## Discussion

The study aimed to assess overall patient experiences and satisfaction with telemedicine services for outpatient and day rehabilitation clinics at our rehabilitation hospital since this was a new service that started shortly after the declaration of the COVID-19 public health emergency. We also examined the effectiveness and satisfaction of delivering multidisciplinary care (physician and rehabilitation therapists) *via* telehealth.

Surveys were completed by 157 respondents representing 6.3% of those who received the email invitation for consent and online survey. We hypothesize several reasons that may have contributed to the low response rate such as technical issues with email distribution (e.g., emails registered as spam/junk), lack of participant incentive for study participation (e.g., payment, raffle prize), or a formal consenting process requiring several steps to participate. However, the survey responses of those 157 participants provide a framework of information on their rehabilitation telehealth experiences and springboard for further research capturing larger sample sizes and broader populations.

Overall, the survey respondents reported a high degree of ease, satisfaction, and effectiveness with their telehealth experience. While about 2/3^rd^ of the respondents had not previously used telehealth for their care at our rehabilitation hospital's clinics, the majority had familiarity with technology for videoconferencing for other purposes (e.g., work, family) or telehealth at other medical centers. As such, the survey respondents were generally comfortable with videoconferencing and found the telehealth web platform easy to use. As the majority of the survey respondents (69.4%) were over the age of 50 years, with about 25% being 70–89 years old, these findings support that older individuals can have high degrees of comfort and familiarity with videoconferencing as well as individuals reflecting younger generations. Similarly, Bhuva et al. found that older individuals in their PM&R practice for spine disorders readily embraced telehealth and technology with positive experiences such that 83.2% of patients 60 years and older were very satisfied with their telemedicine appointment and that most patients (87%) did not have any issues during the telemedicine encounter (7). In a study of adults with disabilities in Australia receiving telehealth allied health care, more than half (47%–67%) reported the telehealth technology being easy to use, effective, and were happy with the privacy and safety (27). Ensuring the ease and comfort of use of telehealth is important for facilitating its use in older populations and those with musculoskeletal, neurological, and chronic conditions or other disabilities who may be likely to receive rehabilitation services and may have unique and complex needs due to motor and cognitive impairments. Moreover, in one study, those with chronic neurological disorders attending PM&R or subspecialty neuro-urology clinics had high rates of perceived difficulty and burden in attending in-person clinic visits due to transportation difficulties, impaired mobility, and changes in daily schedule, and required family/caregiver assistance (28). For many of these patients, telehealth has provided a vital option for access and continuity of care, whether for maintaining outpatient care or after discharge from inpatient rehabilitation hospitalization, particularly in the pandemic.

Ratings for the effectiveness and satisfaction of telehealth visits were overall positive and with high degrees of satisfaction for the full rehabilitation cohort. Survey respondents felt comfortable and safe in the virtual care model and found that they could effectively explain and demonstrate their symptoms. Overall levels of satisfaction with the telehealth experience were high with 79.5% reporting being “very satisfied.” Our findings are in support of other recent telehealth patient satisfaction surveys in outpatient rehabilitation settings published during the pandemic. Studies from single center, outpatient musculoskeletal and spine clinics in the United States and Italy report excellent patient satisfaction in addressing clinical care needs, communication, and recommendations, with some studies reporting this positivity in ∼92%–98% of respondents (7, 13, 29). Patient satisfaction was also high in an academic medical center telemedicine cancer rehabilitation program with patients seen by physiatrists as 94.8% reporting a good experience (18) and in a pilot study of telemedicine for spinal cord injury (16). In our study, on average, 79.8% of survey respondents were “very satisfied” with the effectiveness, comfort, safety, and privacy of their telehealth visits and 78.5% were “very satisfied” with their ability to explain and demonstrate symptoms and for the provider's ability to pay attention, complete assessments, and provide recommendations. While still quite high, these rates are slightly lower than other studies reporting ∼95% satisfaction, and it is possible that this difference may relate to the greater heterogeneity of clinical diagnostic conditions in our rehabilitation patient population studied (i.e., multiple diagnoses vs. restricted to spine or musculoskeletal clinics) or differences in survey questions or timing of survey administration in the pandemic. Interestingly, we did not detect any significant differences in experiences and satisfaction with telehealth across the different clinical diagnostic categories (musculoskeletal conditions, chronic pain, localized pain, neurological conditions, and Parkinson's disease and movement disorders). All patient groups expressed positive feedback and experiences with their telehealth visits. Our study, along with others, support the feasibility, effectiveness, and satisfaction of telemedicine in outpatient rehabilitation settings as well as its use in a variety of conditions for which people seek physiatry care.

The perceived benefits of telehealth for rehabilitation settings and populations in our study and other literature reflect several common themes – reduced travel needs, decreased burden compared to in-person visits as related to mobility or pain issues, increased safety related to COVID risk, improved convenience and more cost-effective. Decreasing burden for travel related to distance and expense, but also for people with impaired motor function, cognitive difficulties, and/or pain, those who have equipment needs, or those who may rely on caregivers for transportation and help is critical for optimizing telehealth use for needed rehabilitation services and appointments for people with disabilities and chronic conditions. In one study, adoption of telehealth and telerehabilitation for musculoskeletal conditions during the pandemic was associated with cost-savings of £38.57 per patient primarily due to reduced travel needs for visit (12), and the use of school-based tele-physiatry for children living in rural and underserved communities in Northern California resulted in an average cost savings of $100 per clinic to the payer due to reducing physician mileage reimbursement (30). Future health economic studies of telehealth for physiatry and rehabilitation services will be needed. One such randomized clinical trial comparing cost-utility and cost-effectiveness of in-person and telemedicine treatment for chronic low back pain is underway (31).

A unique aspect of our study was the examination of viewpoints regarding telehealth care in multi- or inter-disciplinary team rehabilitation settings represented by a subset of the survey participants. The integrated and coordinated team models of physician and rehabilitation therapists (physical therapy, occupational therapy, speech language pathology), whether synchronously or asynchronously delivered at the time of the appointment, were viewed positively. For the synchronously delivered care in the Parkinson's disease and movement disorders visits, the rehabilitation therapists were all in the same “Webex room” performing the telehealth assessment with the patient; they were joined virtually by the physician, advanced practice provider, and nurse, in addition to a team “huddle” during the visit. In the asynchronously delivered model such as in the pain management center, the therapist and physician virtual visits occurred separately but on the same date with communication occurring between team members. Reported benefits of the virtual team care models included receiving multiple perspectives and recommendations, only needing to explain their concerns once, feeling like an integrated member of their healthcare team, having a comprehensive and holistic team approach, and effectively addressing physical and mental health symptoms, and allowing for team communication. The majority of the respondents (54%) had neurological conditions, including Parkinson's disease, Lewy body dementia, Huntington's disease, and other neurological disorders. Although not specifically delivered in rehabilitation or in multidisciplinary settings, there has been growing evidence for the use of telemedicine for Parkinson's disease in clinical neurological care and research including its use preceding the pandemic though with heightened response to COVID-19 (1, 4, 32–35), as well as study of its use for Huntington's disease pre-pandemic (36), in relation to COVID-19 (37, 38), and in two settings of multidisciplinary care (39, 40). Those with chronic pain disorders who often require a multi-disciplinary approach may be well-served with virtual telehealth team care models. Baadiou et al. surveyed rehabilitation team members (physician specialists in rehabilitation medicine, psychologists, physical therapists, and occupational therapists) from a tertiary care pain center regarding telehealth interdisciplinary care delivery in the pandemic and identified key topics regarding videoconference methods, interdisciplinary team work, systems, and efficiencies; the clinicians endorsed that the quality of the pain rehabilitation program could be maintained *via* telehealth with new opportunities brought on by the pandemic (21). Findings from our study support that integrated, person-centered care can be achieved through telehealth. Future studies with larger cohorts as well as prospective studies will enable us to further refine telemedicine team care and apply this to broader rehabilitation populations and settings.

Strengths of this study were the broad recruitment of telehealth participants receiving care at Shirley Ryan AbilityLab which resulted in wide age distribution and representation of various medical conditions and diagnostic categories. Our use of the EDW to link telehealth visit information to the survey email and with electronic medical record diagnoses allowed us to achieve diagnoses and relevant clinical information in a HIPAA-compliant and unbiased manner. In addition, the survey was created with input from a diverse team of clinicians who are physicians and therapists, researchers, data scientists, and administrators and included a range of questions that addressed experience with telehealth and technology, effectiveness and satisfaction with telehealth visit, safety and privacy, and benefits. We were also able to capture feedback on implementing the multidisciplinary care models, which were previously done in-person pre-pandemic, *via* telehealth. Limitations include that our study was based on a single institution which may limit the generalizability of findings to other physiatry practices and settings. However, similar positive results have been reported in other studies coming from single institutions or clinics (7, 13, 18). Respondents to our survey were primarily White, non-Hispanic, and highly educated and thus, future studies are needed to ascertain telehealth feedback from minority and other populations. Compared to other telehealth studies, our sample size was small, though our study design distributed these surveys over a longer duration, an 8-month period, in the pandemic. With small samples as in our survey results, survey responses may reflect sampling bias and also limit generalizability. Surveys in general may be affected by recall bias, though only a small percentage (5%) had visits that predated the survey launch by 1–6 months. Furthermore, in online, web-based studies as well as with technology-focused research, it is plausible that those patients who responded, by nature of completing the online informed consent form and web-based survey, were more comfortable with technology and digital environments, thereby providing more positive responses.

Future directions include continued refinement of telehealth services with protocols for set up for both the provider and patient, conducting rehabilitation examinations, and optimizing care delivery and coordination, and its evidence base for people with disabilities and acute or chronic conditions requiring physiatry and rehabilitation evaluations and services. Recently, there have been protocols proposed for virtual examinations in PM&R settings and for rehabilitation populations such as for physical examination of orthopedic issues, musculoskeletal system (e.g., shoulder, hand, back), neurological and movement disorders (20, 41–47). In addition, recommendations for chronic pain management during and for after the COVID-19 pandemic have been developed by multidisciplinary experts in pain management using a modified Delphi approach and suggesting needs regarding organizational changes, structuring careful diagnostic and therapeutic pathways, and applying new technologies in pain medicine (48). Education and training programs for PM&R (physiatry) and other disciplines have adapted in the pandemic and utilization of telehealth to provide and incorporate curriculum geared to virtual physical examinations, patient safety considerations, and trainee fellowship changes (e.g., in pain management programs and musculoskeletal education) (42, 43, 49, 50).

Gaps and disparities, however, remain regarding access to telehealth services including internet, computers or digital systems, and comfort and literacy with navigating digital platforms. Greater understanding of the barriers that limit the access to or continuation of telehealth use among patients and healthcare providers is paramount to successful implementation and sustainment of telehealth. Considerations for insurance coverage and reimbursement, cross-state telehealth or universal licensure, and policies to provide telehealth services beyond the COVID-19 public health emergency are also critical topics. Prospective studies will be needed to assess the long-term effects of telehealth in rehabilitation populations on outcomes, functional abilities, and quality of life.

## Conclusion

In conclusion, the findings reported in the current study reveal that telemedicine delivery is feasible in our rehabilitation setting for outpatient and day rehabilitation program clinics and that the majority of survey respondents view these telehealth services as easy to use, effective, safe, and beneficial to their care. This satisfaction was high across multiple diagnoses including musculoskeletal disorders, neurological conditions, and pain issues. In addition, patient care delivered by multidisciplinary care teams was effective in the telehealth format. Our findings will help inform telehealth practices at our rehabilitation hospital settings, continued quality improvement and best practices, and use of innovative care delivery. Further study is needed to understand the generalizability of our findings to broader demographic populations as well as the long-term impact on patient care and healthcare system outcomes.

## Data Availability

The raw data supporting the conclusions of this article will be made available by the authors, without undue reservation.
