# Supplementary material for: Patient experiences receiving rehabilitation care via telehealth: Identifying opportunities for remote care
Source: Front Rehabil Sci. 2023 Feb 2;4:1049554. doi: 10.3389/fresc.2023.1049554 (PMC9932031; doi:10.3389/fresc.2023.1049554)
Supplement: Supplementary file 1 [file Datasheet1.pdf]

Assessing Patients' Experiences with Telehealth Services at Shirley Ryan AbilityLab

Instructions

This survey will ask you about your experiences with telehealth services at Shirley Ryan AbilityLab.
Please complete each question to the best of your knowledge. Your input and feedback on our telehealth services can help us understand them better and improve telehealth services and care. You may ask for help from others when answering these questions, but this survey is meant for patients to complete, either alone or with their parents/family/care partners/etc.
If you have any questions about the survey, please contact redcap@sralab.org.
Thank you.

A. General information

Please check who is completing this survey:

- ◯ Patient mostly
- ◯ Caregiver mostly
- ◯ Patient and caregiver in equal amounts

Please tell us the following information about yourself: (patient)

Age (in years)

- ◯ 0-2
- ◯ 3-7
- ◯ 8-12
- ◯ 13-17
- ◯ 18-29
- ◯ 30-39
- ◯ 40-49
- ◯ 50-59
- ◯ 60-69
- ◯ 70-79
- ◯ 80-89
- ◯ 90 and above

Sex

- ◯ Female
- ◯ Male
- ◯ Prefer not to answer

Ethnicity

- ◯ Hispanic or Latino
- ◯ Not Hispanic or Latino
- ◯ Prefer not to answer

Race (check all that apply)

- ☐ White
- ☐ Black or African American
- ☐ Asian
- ☐ Native American or American Indian
- ☐ Native Hawaiian or Pacific Islander
- ☐ Other
- ☐ Prefer not to answer

Education, highest degree or level of school you have completed? If currently enrolled, highest degree received

- ◯ Less than high school degree
- ◯ High school degree or equivalent
- ◯ Some college but no degree
- ◯ Associate degree
- ◯ Bachelor (college) degree
- ◯ Graduate degree

Distance from your home to the location of your Shirley Ryan AbilityLab telehealth visit	◯ 0-5 miles<br>◯ 6-10 miles<br>◯ 11-15 miles<br>◯ 16-20 miles<br>◯ Greater than 20 miles
Please select your Insurance provider:	◯ Medicare<br>◯ Medicaid<br>◯ Commercial insurer (e.g., Aetna, Blue Cross Blue Shield, Humana, United Health)<br>◯ Don't know<br>◯ Prefer not to answer
How did you hear about us? Please check all that apply.	☐ Referred by my physician<br>☐ Referred by friend or family member<br>☐ Shirley Ryan AbilityLab website (www.sralab.org)<br>☐ Social media (Facebook, Instagram, LinkedIn)<br>☐ Radio or television advertisement<br>☐ News articles<br>☐ Web Search (Google, Bing , DuckDuckGo)<br>☐ Chicago Marathon<br>☐ Community event<br>☐ Virtual free screening program at Shirley Ryan AbilityLab<br>☐ Other Shirley Ryan AbilityLab program (inpatient, Adaptive fitness center)

B. General information about your telehealth visit at Shirley Ryan AbilityLab

Did you have a telehealth visit at Shirley Ryan AbilityLab?

- ◯ Yes
- ◯ No

Please tell us why. Check all that apply.

- ☐ Not interested
- ☐ Preferred to wait until in-person visit available
- ☐ Concerns about using telehealth
- ☐ Lack of access to phone
- ☐ Lack of access to computer
- ☐ Lack of internet
- ☐ Other

Please specify.

____

Month of first telehealth visit:

- ◯ January
- ◯ February
- ◯ March
- ◯ April
- ◯ May
- ◯ June
- ◯ July
- ◯ August
- ◯ September
- ◯ October
- ◯ November
- ◯ December

Please select the location of the telehealth visit:

[List of locations omitted for this example survey]

Please choose a Shirley Ryan AbilityLab Physician.

[List of physicians omitted for this example survey]

Please select your visit type:

- ◯ First visit with this provider (new patient appointment)
- ◯ Follow up visit with this provider

Please select the modality you used for your telehealth visit:

- ◯ Phone only (no video)
- ◯ Videoconference

Please tell us what device you used. Check all that apply.	◯ Computer<br>◯ iPad/tablet<br>◯ Smart phone<br>◯ Other
Did anything prevent you from completing the entire telehealth visit as planned?	◯ Yes<br>◯ No
Please tell us what happened.	◯ Audio failure<br>◯ Video failure<br>◯ Both audio and video failed<br>◯ Other
Please specify.	______________________________
Have you previously utilized telehealth services for your care at Shirley Ryan AbilityLab?	◯ Yes<br>◯ No<br>◯ Not sure
For what reasons?	◯ Physician appointment<br>◯ Rehabilitation therapy appointment<br>◯ Both<br>◯ Other
Have you previously utilized telehealth services for your care at other hospitals or medical centers?	◯ Yes<br>◯ No<br>◯ Not sure
When was your last telehealth visit prior to this visit?	◯ Less than one week ago<br>◯ Within the past month<br>◯ Greater than a month ago
Do you currently use videoconference (Zoom, Webex, Facetime, etc.) for other purposes such as work or with family/friends?	◯ Yes<br>◯ No
Please rate how often you use videoconference:	◯ Very frequently<br>◯ Somewhat frequently<br>◯ Occasionally<br>◯ Somewhat infrequently<br>◯ Very infrequently
Please rate your comfort with using videoconference:	◯ Very comfortable<br>◯ Somewhat comfortable<br>◯ Neutral<br>◯ Somewhat uncomfortable<br>◯ Very uncomfortable
Did someone in your location help you during your telehealth visits?	◯ Yes<br>◯ No

Who? Check all that apply	☐ Spouse/partner<br>☐ Parent<br>☐ Grandparent<br>☐ Child<br>☐ Other family member<br>☐ Friend<br>☐ Caregiver (non-family)<br>☐ Other (specify relationship)
Please specify relationship.	__________________________

C. Please tell us about your experience getting ready for this telehealth visit and using the Webex platform. Please rate the following.

	Very helpful	Helpful	Neutral	Unhelpful	Very unhelpful	Not applicable
Instructions for setting up the system before the telehealth visit	○	○	○	○	○	○
Technical support before the telehealth visit	○	○	○	○	○	○
Technical support, if needed, at the time of the visit	○	○	○	○	○	○

	Very easy	Fairly easy	Average	Fairly difficult	Very difficult
Ease of connecting to the visit	○	○	○	○	○
Ease of using the telehealth system, once connected	○	○	○	○	○

	Very good	Good	Fair	Poor	Very poor
Audio quality	○	○	○	○	○
Video quality	○	○	○	○	○

D. Your experience during this telehealth visit examination. Rate your satisfaction with the following.

	Very satisfied	Somewhat satisfied	Neutral	Somewhat dissatisfied	Very dissatisfied
Effectiveness of getting your care through telehealth	○	○	○	○	○
Comfort level in being evaluated and treated through telehealth	○	○	○	○	○
Feeling of physical safety (e.g., moving, walking) during the telehealth visit	○	○	○	○	○
Feeling that your privacy was safe during the telehealth visit	○	○	○	○	○
Your ability to explain and demonstrate your symptoms adequately during telehealth	○	○	○	○	○
The ability of the provider to pay full attention to you during the telehealth visit	○	○	○	○	○
The ability of the provider to complete his or her assessments for your condition with telehealth	○	○	○	○	○
The ability of the provider to give you the necessary recommendations for my condition through telehealth	○	○	○	○	○
Your ability to complete provider's assessments without someone else (e.g., family member, caregiver) present to help you with the exam	○	○	○	○	○

E. Your overall impressions of the telehealth visit. Rate the following:

Your overall level of satisfaction with the telehealth experience

◯ Very satisfied
◯ Somewhat satisfied
◯ Neutral
◯ Somewhat dissatisfied
◯ Very dissatisfied

My experience with telehealth was:

◯ Better than I expected
◯ About the same as I expected
◯ Worse than I expected

	Very likely	Somewhat likely	Neutral	Somewhat unlikely	Very unlikely
How likely are you to continue with telehealth visits, if available as part of your routine care, even after the COVID-19 pandemic?	◯	◯	◯	◯	◯
How likely are you to tell your other healthcare providers (e.g., primary care physician, other physicians, rehab therapy team) that telehealth worked well for you?	◯	◯	◯	◯	◯
How likely are you to recommend telehealth to others such as friends or relatives?	◯	◯	◯	◯	◯

Have you received in-person clinical care for your condition in the past at Shirley Ryan AbilityLab?

◯ Yes
◯ No
◯ Not sure

How did it compare to your telehealth experience at Shirley Ryan AbilityLab?

◯ Much more effective
◯ Somewhat more effective
◯ Equally effective
◯ Somewhat less effective
◯ Much less effective

What were the benefits of this telehealth visit? Check all that apply

☐ Less time away from work or home duties
☐ Able to do from my home or location of choice
☐ No need to commute or travel to appointment
☐ Less cost for travel (no gas mileage, taxi fare, flight, etc)
☐ Other (please explain)

Please explain "Other":

____

What would make your telehealth visit experience better or more effective? Check all that apply.	☐ Better audio connection<br>☐ Better video connection<br>☐ More space available in my location to demonstrate my issue to the provider (physician or therapist)<br>☐ Family member or caregiver to help me during the session (e.g., to position phone, computer camera)<br>☐ Other (please explain)<br>☐ No changes needed - my telehealth visit went well
Please explain "Other":	__________________________________

F. Your experience with using telehealth in our interdisciplinary team care clinics. Rate the following:

Have you received evaluation and care within our telehealth interdisciplinary team clinic? In this type of clinic, you were seen by the physician and the Physical Therapist (PT), Occupational Therapist (OT) and/or Speech Language Pathologist (SLP) (or a combination of PT, OT, SLP) at the same telehealth appointment date.

◯ Yes
◯ No

	Strongly agree	Somewhat agree	Neutral	Somewhat disagree	Strongly disagree
I received multiple perspectives and recommendations for my symptoms	◯	◯	◯	◯	◯
I only needed to explain my concerns once	◯	◯	◯	◯	◯
I felt like an integrated member of my healthcare team	◯	◯	◯	◯	◯
I appreciated the teams comprehensive and holistic approach to my condition	◯	◯	◯	◯	◯
My concerns about physical symptoms were effectively addressed by the team	◯	◯	◯	◯	◯
My concerns about mental health symptoms were effectively addressed by the team	◯	◯	◯	◯	◯
I felt like the team members effectively communicated with each other	◯	◯	◯	◯	◯
I felt like the team members effectively communicated their findings and recommendations to me	◯	◯	◯	◯	◯
The time spent with each discipline (PT, OT, SLP, physician) was adequate	◯	◯	◯	◯	◯
The team approach allowed me to address topics that are not always covered in my visits with a single physician	◯	◯	◯	◯	◯
I would recommend my experience at the Shirley Ryan AbilityLab interdisciplinary team care to others (friends, relatives, etc.)	◯	◯	◯	◯	◯
This type of interdisciplinary team evaluation worked well in a telehealth format	◯	◯	◯	◯	◯

Other comments ______________________

G. Additional Comments

Comments ____________________________________
